# P-449. Divergent Microbial Profiles in Pediatric Fecal and Urinary Samples: The Role of Urinary Tract Infections

**DOI:** 10.1093/ofid/ofaf695.664

**Published:** 2026-01-11

**Authors:** Ji Hyen Lee, Jung Won Lee

**Affiliations:** Ewha Womans University Seoul Hospital, Seoul, Seoul-t'ukpyolsi, Republic of Korea; Ewha Womans University Seoul Hospital, Seoul, Seoul-t'ukpyolsi, Republic of Korea

## Abstract

**Background:**

Urinary tract infection (UTI) is the most common bacterial infection in infants, often resulting from fecal uropathogens. Lactobacillus strains have shown potential in preventing E. coli infections and inhibiting urogenital pathogens. The urinary microbiome plays a crucial role in bladder homeostasis, and its dysbiosis is linked to UTIs and lower urinary tract dysfunction. However, studies on pediatric populations remain limited. This study investigates differences in gut and urinary microbiome composition between febrile UTI patients and healthy controls.

**Figure 1.**
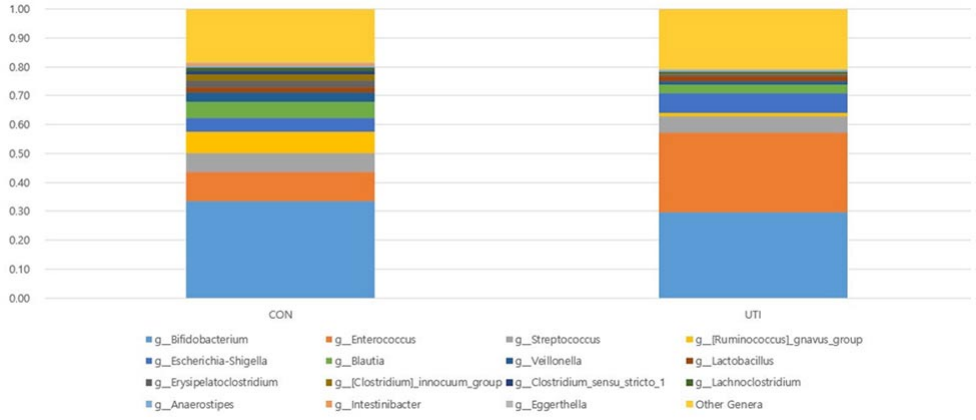
Relative abundance of bacterial genera in stool samples from control and urinary tract infection groups

**Figure 2.**
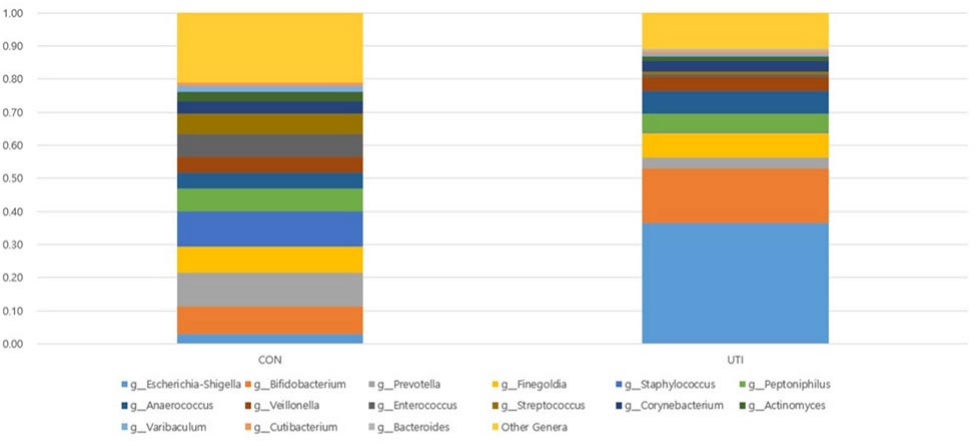
Relative abundance of bacterial genera in urine samples from control and urinary tract infection groups

**Methods:**

From January to December 2023, 59 children were analyzed, including 43 healthy controls and 16 UTI patients. The control group consisted of children visiting clinics for routine vaccinations, while the UTI group included febrile patients ( >38°C) with pyuria and bacteriuria. Clinical variables assessed included age, sex, fever duration, feeding history, birth details, antibiotic use, stool consistency, and urine culture results. Blood tests and imaging studies were performed for the UTI group. Stool and urine samples underwent 16S rRNA gene sequencing (V3–V4 region) using the Illumina MiSeq platform. Shannon diversity indices and relative abundances at the genus and species levels were analyzed using the Mann-Whitney U and Kruskal-Wallis tests.

**Figure 3.**
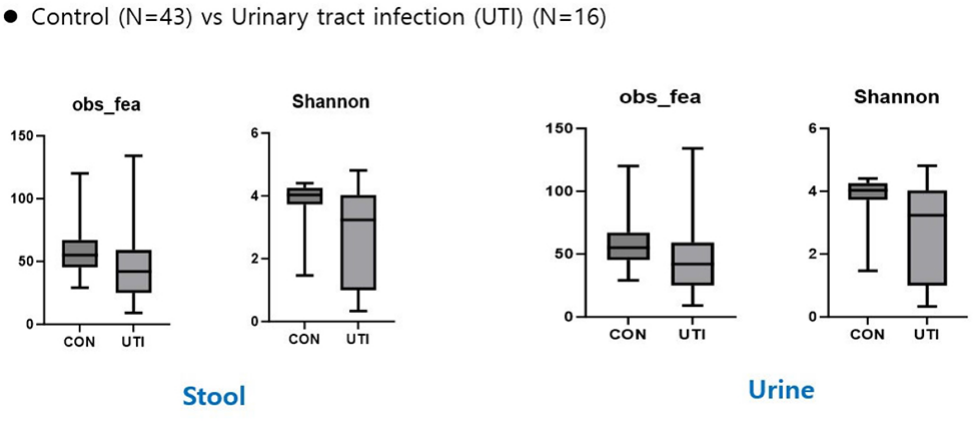
Alpha diversity of microbial diversity observed in stool and urine between the control group and the urinary tract infection group

**Figure 4.**
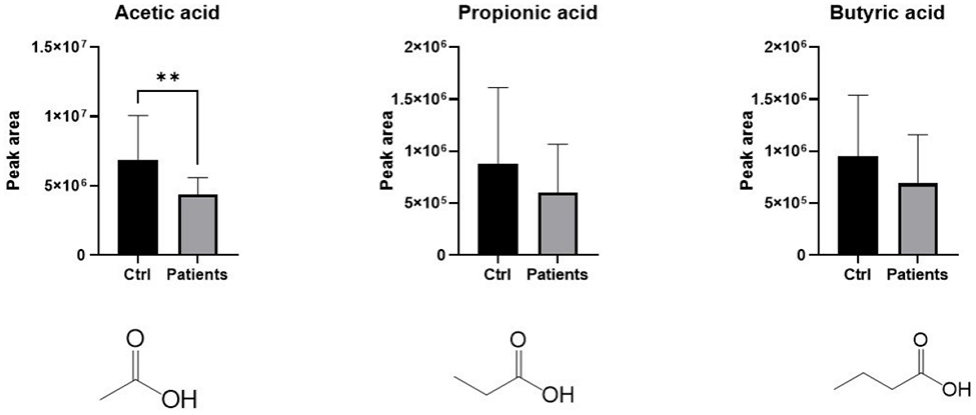
Reduction of short-chain fatty acids in pediatric urinary tract infection patients

**Results:**

The mean age was 14.7 months in the control group and 26.8 months in the UTI group. Alpha diversity analysis showed significantly lower microbial diversity in the UTI group compared to controls (P = 0.037) (Figures 1-3). Additionally, short-chain fatty acids (SCFAs), including acetic acid, propionic acid, and butyric acid, were significantly reduced in the feces of UTI patients (Figure 4).

**Conclusion:**

Significant differences in microbiome diversity and composition were observed between pediatric UTI patients and healthy controls. The pediatric urinary microbiome, particularly in infants, is an emerging research area. Further studies are needed to elucidate its unique characteristics across different pediatric populations.

**Disclosures:**

All Authors: No reported disclosures

